# Systematic Review on S-ICD Lead Extraction

**DOI:** 10.3390/jcm12113710

**Published:** 2023-05-27

**Authors:** Riccardo Vio, Enrico Forlin, Viktor Čulić, Sakis Themistoclakis, Riccardo Proietti, Paolo China

**Affiliations:** 1 Department of Cardiothoracic, Vascular Medicine and Intensive Care, Dell’Angelo Hospital, 30174 Mestre-Venice, Italy; riccardo.vio.1@gmail.com (R.V.); forlinenrico1@gmail.com (E.F.); themistoclakis@yahoo.it (S.T.); paolo.china@gmail.com (P.C.); 2 Department of Cardiac, Thoracic and Vascular Sciences and Public Health, University of Padua, 35128 Padua, Italy; 3 School of Medicine, University of Split, 21000 Split, Croatia; viktor.culic@st.t-com.hr; 4 Department of Cardiology and Angiology, University Hospital Centre Split, 21000 Split, Croatia; 5 Liverpool Centre for Cardiovascular Science, University of Liverpool and Liverpool Heart and Chest Hospital, Liverpool L8 7TX, UK

**Keywords:** subcutaneous implantable cardioverter defibrillator, S-ICD, ICD, extraction, explantation, infection

## Abstract

Background and purpose: Subcutaneous implantable cardioverter defibrillators (S-ICDs) have emerged in recent years as a valid alternative to traditional transvenous ICDs (TV-ICDs). Therefore, the number of S-ICD implantations is rising, leading to a consequent increase in S-ICD-related complications sometimes requiring complete device removal. Thus, the aim of this systematic review is to gather all the available literature on S-ICD lead extraction (SLE), with particular reference to the type of indication, techniques, complications and success rate. Methods: Studies were identified by searching electronic databases (Medline via PubMed, Scopus and Web of Science) from inception to 21 November 2022. The search strategy adopted was developed using the following key words: subcutaneous, S-ICD, defibrillator, ICD, extraction, explantation. Studies were included if they met both of the following criteria: (1) inclusion of patients with S-ICD; (2) inclusion of patients who underwent SLE. Results: Our literature search identified 238 references. Based on the abstract evaluation, 38 of these citations were considered potentially eligible for inclusion, and their full texts were analyzed. We excluded 8 of these studies because no SLE was performed. Eventually, 30 studies were included, with 207 patients who underwent SLE. Overall, the majority of SLEs were performed for non-infective causes (59.90%). Infection of the device (affecting either the lead or the pocket) was the cause of SLE in 38.65% of cases. Indication data were not available in 3/207 cases. The mean dwelling time was 14 months. SLEs were performed using manual traction or with the aid of a tool designed for transvenous lead extraction (TLE), including either a rotational or non-powered mechanical dilator sheath. Conclusions: SLE is performed mainly for non-infective causes. Techniques vary greatly across different studies. Dedicated tools for SLE might be developed in the future and standard approaches should be defined. In the meantime, authors are encouraged to share their experience and data to further refine the existing variegated approaches.

## 1. Introduction

Since their development, Implantable Cardioverter Defibrillators (ICDs) have been a fundamental part of the primary and secondary prevention of sudden cardiac death (SCD) [1]. Although transvenous ICDs (TV-ICDs) are still the standard of care, their long-term use is associated with a variety of device-related complications, such as infections or lead displacement and rupture, in some cases requiring surgical revision or even the extraction of the entire device [2,3]. Subcutaneous ICDs (S-ICDs) thus became a valid alternative to reduce the occurrence of adverse events related to the presence of intravenous leads, especially in young patients with a long life expectancy, normal heart, and no need for pacing or cardiac resynchronization therapy, at increased risk of infections and with limited vascular access [4,5]. Since S-ICDs have consistently been proved to be effective in terminating malignant arrhythmias, the implantation of such devices is rising. In parallel with the growing number of S-ICDs implanted, there is an absolute increase in S-ICD-related complications (e.g., infection) that may require complete system removal [6,7,8]. Although transvenous lead explantation (TLE) is a relatively common procedure, with several approaches, dedicated tools and specific guidelines [9], S-ICD lead extraction (SLE) still relies on poor clinical data regarding its technical execution (with simple manual traction as the only established method), efficacy and safety [10,11]. This could be particularly relevant not only for the increasing necessity of extraction procedures, but also for the growing dwelling time of the S-ICDs implanted, typically associated with the development of fibrotic adherences and calcifications around the lead that could interfere with extraction [10]. Thus, the aim of this systematic review is to gather all the available literature on SLE, with particular reference to type of indication and the method used to perform the extraction.

## 2. Materials and Methods

### 2.1. Design

This systematic review was conducted and reported according to Preferred Reporting Items for Systematic Reviews and Meta-Analyses (PRISMA) recommendations [12]. The systematic review was not registered.

### 2.2. Study Selection

Studies were identified by searching electronic databases (Medline via PubMed, Scopus and Web of Science) from inception to 21 November 2022. The literature search used text and relevant indexing to capture data on S-ICD lead extraction. The search strategy adopted was developed using the following key words: subcutaneous, S-ICD, defibrillator, ICD, extraction, explantation. The string adopted for PubMed was (“subcutaneous” OR “S-ICD”) AND (“defibrillator” OR “ICD”) AND (“extraction” OR “explantation”). Only studies in English were included in the review. Studies were included if they met both of the following criteria: (1) inclusion of patients with S-ICD; (2) inclusion of patients who underwent SLE.

### 2.3. Data Extraction

Information was extracted from each included study regarding the (1) baseline characteristics of patients and country where the study was performed; (2) indications for SLE; (3) dwelling time; (4) technique used for SLE; (5) complications; (6) success; and (7) management after SLE. Two authors (R.V. and E.F.) independently extracted data from studies and entered them into the data extraction form. Disagreements were resolved by discussion; if no accord was reached, it was planned that a third author (P.C.) would decide.

### 2.4. Quality Assessment

The JBI tool was used to perform the quality assessment of the included studies [13]. Supplementary Materials S1 reports how studies were rated.

## 3. Results

### 3.1. Study Selection

**Figure 1:**
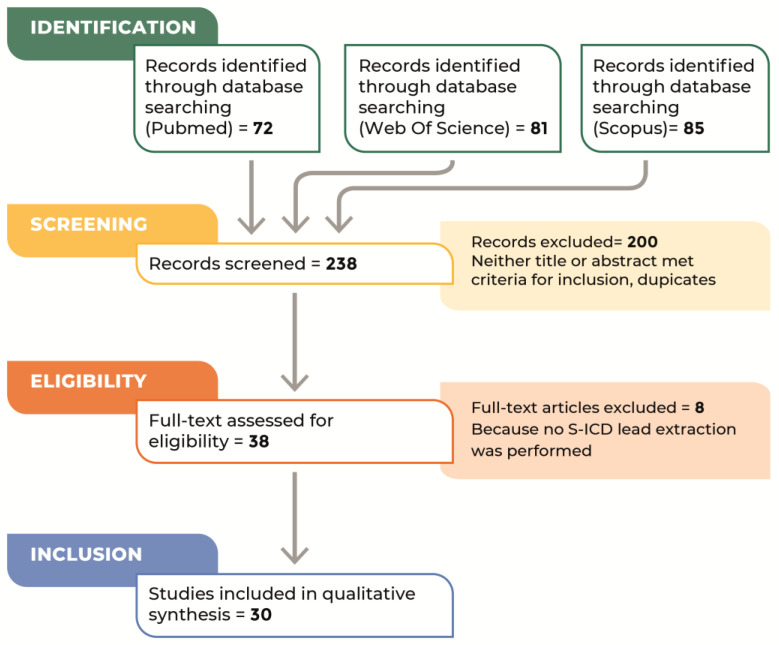
Flow chart of the literature search.

Our literature search identified 238 references (Figure 1). Based on the abstract evaluation, 38 of these citations were considered potentially eligible for inclusion, and their full texts were analyzed in more detail [10,11,14,15,16,17,18,19,20,21,22,23,24,25,26,27,28,29,30,31,32,33,34,35,36,37,38,39,40,41,42,43,44,45,46,47,48,49]. We excluded 8 of these studies because no SLE was performed [10,18,19,21,27,29,46,47]. Eventually, 30 studies were included [10,11,14,15,16,17,20,22,23,24,25,26,28,30,31,32,33,34,35,36,37,38,39,40,41,43,44,45,48,49].

### 3.2. Characteristics of the Studies

Overall, 207 patients were included in this analysis. One patient underwent SLE twice [43]. Of the 30 included studies, the majority (16/30) were retrospective cohort studies [15,16,17,22,23,26,30,33,36,37,38,41,43,44,48,49]. Five were prospective studies [14,31,32,34,35], and the remaining nine were case reports [10,11,20,24,25,28,39,40,45]. The number of patients included in the studies ranged from 1 to 1637. The full study characteristics are summarized in Table 1.

### 3.3. Indications for S-ICD Extraction

Overall, the majority of SLEs were performed for non-infective causes (59.90%). Among these, the occurrence of inappropriate shocks was responsible for 16.91% of total SLEs, necessity for cardiac resynchronization therapy (CRT) was responsible for 8.70%, progression of disease to heart transplantation or left ventricular assist device (LVAD) implantation was responsible for 7.25% and sensing issues were responsible for 4.35%. Infection of the device (affecting either the lead or the pocket) was the cause of SLE in 38.65% of cases. Indication data were not available in 3/207 cases [17,22]. The full list of all the indications is reported in Table 2. 

### 3.4. Dwelling Time

The mean time from implantation to extraction (i.e., dwelling time) of S-ICDs was 14 months, ranging from 30 days [37] to over 8 years [36]. The dwelling time was not available in 10 studies [14,15,16,20,22,23,32,35,38,44]. The majority of studies in which this information was reported showed a mean time of one year or less [24,26,33,36,39,43,45,48]. The dwelling time was particularly short in studies where infections were the indication for more than 50% of SLEs performed [31,36,37,43].

### 3.5. Method Used for SLE

In the studies analyzed, SLE required a two- or three-incision technique, depending on the way the lead was originally inserted and on eventual complications. The first incision was performed generally along the midaxillary line, in order to open the pocket and remove the pulse generator, freeing also the proximal part of the lead. The second incision was then executed on the region of xiphoid apophysis, fundamental to release the suture anchoring the lead and usually exploited as the access to extract the proximal and distal ends of the lead. An additional incision could be performed at the level of the manubriosternal junction in order to reach the distal coil of the lead, cutting eventual sutures that anchored it to the periosteal fascia or releasing it from fibrous tissue. Manual traction was then applied to unthread the lead, generally from subxiphoid access. In some cases, typically characterized by a long dwelling time, fibrosis of the tissues around the lead offered resistance to its removal, requiring the use of a mechanical sheath to relieve it [9,10,18,24,26]. This tool, originally designed for TLE, is manufactured to be slipped around the lead and then, with traction and counter-traction and simultaneous rotation, progressively advanced over the catheter. This technique allows the operator to dilatate and to break off fibrotic tissue along the lead, favoring its removal. Additional treatment could then be executed according to the S-ICD extraction’s indication (e.g., local antimicrobial treatment in case of pocket infection). The extraction technique was described in 11 studies [10,11,17,20,24,25,26,28,36,40,41], a minority of those included in this analysis. Among these studies, 7/11 were case reports [10,11,20,24,25,28,40]. Table 3 reports the methods used for SLE in our study population. 

### 3.6. Complications and Success Rate

There were no periprocedural complications reported during SLE. This point was specifically addressed by the two included studies reporting the greatest numbers of SLEs [17,26]. Pothineni et al. reported that in their cohort of 64 patients who underwent SLE, no complications occurred [17]. The secondary endpoint of the study by Behar et al. [26] included procedural complications, and again, no procedure-related complications were reported. 

Only 1 case of procedural failure occurred among the 207 reported because the lead could definitely not be extracted [26]. 

### 3.7. Management after SLE

Table 4 reports which management options were chosen after SLE, including the percentages of S-ICD or TV-ICD reimplantation.

Of note, in the study by Brouwer et al., one patient underwent a second S-ICD extraction after SLE during subsequent follow-up for recurrent infection [43].

## 4. Discussion

To the best of our knowledge, our study is the first systematic review on SLE. 

According to our systematic review, the majority of SLEs are performed for non-infective causes (59.90%). These data differ from those for TLEs, which are performed mainly for infective causes (52.8%) [50]. Among the non-infective indications for SLE, inappropriate shocks accounted for the most part (16.91%). Moreover, sensing issues accounted for 4.35% of cases. Despite rare cases of undersensing of ventricular fibrillation [17], sensing issues usually include refractory oversensing due to myopotential, P- or T-wave oversensing or R-wave double counting [30]. Eventually, oversensing may lead to inappropriate shocks. Inappropriate shocks are a well-known drawback of S-ICDs, significantly affecting patients’ quality of life [51]. However, recent data showed a consistent reduction in inappropriate shocks by S-ICDs (3.1% in 1 year) with the use of high-rate cutoffs, as well as current generation electrogram filtering and discrimination algorithms [52]. Additionally, exercise tests before S-ICD implantation, especially in patients with Brugada syndrome, may further reduce the incidence of future inappropriate shocks [53]. Given all the abovementioned improvements, we can expect a decrease for such indications in future studies on SLE. 

A necessity for resynchronization therapy or pacing is among other prominent causes for SLE. Even though it usually cannot be predicted at implantation, accurate selection of candidates for S-ICD implantation must be performed to minimize this possible scenario.

The dwelling time was very different in our systematic review on SLE compared to that reported for TLE. In fact, our study showed a mean time from implant to extraction of 14 months, compared to a mean dwelling time of 6.4 years in the ELECTRA study on TLE [50]. This discrepancy can be explained by several factors. First, in the studies included in our systematic review, many authors included patients in which the device was removed within a year and with simple manual traction; these procedures are better referred to as “explantation”, according to the terminology used for transvenous PM/ICDs. Other authors did not mention at all the dwelling time or the approach used for extraction, but we decided to include all studies in order to gather all the available literature in the field. Secondly, S-ICDs have been available since the early 2010s, whereas transvenous PM/ICDs were released on the market several years prior, and long-term follow-up data are available. Lastly, infections on transvenous PM/ICDs may occur as part of a systemic infection/bacteremia with the source of infection coming from a different site. In the case of S-ICDs, infections are primarily local and related to the implantation procedure; therefore, they tend to occur earlier. In fact, we found that the dwelling time was particularly short in studies where infections were the indication for more than 50% of SLEs [31,36,37,43]. 

Regarding techniques for SLE, simple manual traction is the easier way, and it was sufficient to remove S-ICD leads in many of the cases reported (99/207) (Table 3). However, this approach cannot always be pursued due to fibrosis surrounding the parasternal coil. According to our systematic review, in 14/207 cases, tools were used to complete a successful SLE. Patel et al. were the first authors to report the successful use of a rotating mechanical dilator sheath (TightRail, Spectranetics) for SLE [28]. Later on, Allison et al. replicated an SLE using the rotating mechanical dilator sheath (TightRail, Spectranetics), but also with the aid of a bulldog lead extender (Cook Medical) [20]. Migliore et al. reported the use of a non-powered mechanical dilator sheath (LR-TSS-11.0, Cook Medical) to disrupt fibrotic adhesions around the coil and the distal tip of the S-ICD lead [10]. The lead was then retracted into the sheath and successfully extracted in the absence of any complications. These studies highlight the absence of a common strategy and of dedicated tools for SLE, which might be developed in the future.

After SLE, a large number of patients (75/207) underwent TV-ICD reimplantation (Table 4). A minor part of the cohort underwent S-ICD reimplantation (25/207). Of note, one patient was extracted and reimplanted twice with an S-ICD because of recurrent local infection [43]. This event highlights the importance of a waiting period before reimplantation to allow complete healing from infection. In this scenario, wearable ICDs may act as a bridge therapy. In the study by Van der Stuijt et al. [23], the authors provided a patient with a wearable ICD after SLE for 3 months, allowing the resolution of infection with antibiotics before S-ICD reimplantation.

### Limitation

Our study has several limitations. The results are based primarily on retrospective studies and case reports, and we acknowledge the risk of overlapping cohorts among different studies. Furthermore, as discussed above, no clear distinction was made between explantation and extraction, and all cases of S-ICD lead removal were collectively considered as SLE. Finally, we could not exclude possible bias due to overlapping cohorts between some of the included studies.

## 5. Conclusions

According to our systematic review, the causes for SLE are primarily non-infective, and the dwelling time seems to be lower compared to that in the literature data on TLE. Techniques vary greatly across different studies, including manual traction and the use of either rotational or non-powered mechanical sheaths. Dedicated tools for SLE might be developed in the future, and standard approaches should be defined. In the meantime, authors are encouraged to share their experience and data to further refine the existing variegated approaches. 

## Figures and Tables

**Table 1:** Characteristics of the included studies.

Author, Year of Publication	Design	Study Population, Country	No. of Pts Who Underwent S-ICD Lead Extraction
Gold 2022 [14]	Prospective cohort study	1637 S-ICD pts (Post Approval Study), U.S.	5
Giacomin 2022 [15]	Retrospective cohort study	36 consecutive S-ICD pts after TLE, Italy	4
Russo 2022 [16]	Retrospective cohort study	317 consecutive S-ICD pts (besides 290 TV-ICD), Italy	1
Pothineni 2022 [17]	Retrospective cohort study	64 S-ICD explanted pts, U.S.	64
Migliore 2021 [10]	Case report	1 S-ICD pt after TLE, Italy	1
Allison 2021 [20]	Case report	1 S-ICD pt, U.S.	1
Chung 2021 [22]	Retrospective cohort study	144 S-ICD pts, Germany	11
Van der Stuijt 2021 [23]	Retrospective cohort study	72 S-ICD pts who underwent elective PGR, Netherlands	1
Gutleben 2020 [24]	Case report	1 S-ICD pt with BrS, Germany	1
Mitacchione 2020 [25]	Case report	1 S-ICD pt with DCM, Italy	1
Behar 2020 [26]	Retrospective cohort study	32 S-ICD explanted pts, France	32
Patel 2020 [28]	Case report	1 S-ICD pt with DCM, US	1
Noel 2020 [30]	Retrospective cohort study	108 S-ICD pts, France	6
Schaller 2019 [31]	Prospective cohort study	13 pts (3 PM, 9 TV-ICD, 1 S-ICD) presenting for CIED extraction due to infection, U.S.	1
Migliore 2019 [32]	Prospective cohort study	101 S-ICD pts, Italy	2
Ip 2019 [11]	Case report	1 S-ICD pt with ICM, U.S.	1
Migliore 2019 [33]	Retrospective cohort study	44 S-ICD pts with AC, Italy	1
Orgeron 2018 [34]	Prospective cohort study	29 S-ICD pts with AC, U.S./Italy	3
Viani 2019 [35]	Prospective cohort study	229 pts who underwent TV-ICD extraction and subsequent S-ICD or TV-ICD implantation, Italy	3
Nakhla 2018 [36]	Retrospective cohort study	21 pts who underwent Medtronic SQC extraction, U.S.	21
Quast 2018 [37]	Retrospective cohort study	118 S-ICD pts, Netherlands	10
Sponder 2018 [38]	Retrospective cohort study	236 S-ICD pts, Austria	4
Clacaianu 2017 [39]	Case report	1 S-ICD pt, France	1
Morani 2017 [40]	Case report	1 S-ICD pt with BrS, Italy	1
Frommeyer 2016 [41]	Retrospective cohort study	24 S-ICD pts with electrical heart disease or idiopathic VF, Germany	1
Brouwer 2016 [43]	Retrospective cohort study	123 S-ICD pts, Netherlands	7 *
Boersma 2016 [44]	Retrospective cohort study	866 S-ICD pts, International	1
Frommeyer 2015 [45]	Case series	93 S-ICD pts, Germany	6
Theuns 2015 [49]	Retrospective registry	55 S-ICD pts, Europe/New Zealand	5
Jarman 2013 [48]	Retrospective registry	111 S-ICD pts, U.K.	10

S-ICD, subcutaneous implantable cardioverter defibrillator; TLE, transvenous lead extraction; TV-ICD, transvenous implantable cardioverter defibrillator; PGR, pulse generator replacement; Brs, Brugada Syndrome; DCM, dilated cardiomyopathy; PM, pacemaker; CIED, cardiovascular implantable electronic device; ICM, ischemic cardiomyopathy; AC, arrhythmogenic cardiomyopathy; SQC, subcutaneous shocking coils; pt, patient; pts, patients. * 1 pt underwent extraction twice.

**Table 2:** Indications for SLE.

Indication for SLE	Total Population, *n* = 207
Non-infective, *n* (%)	124 (59.90%)
-Inappropriate shocks, *n* (%)	35 (16.91%)
-Necessity for CRT, *n* (%)	18 (8.70%)
-Heart transplantation/LVAD, *n* (%)	15 (7.25%)
-Sensing issues, *n* (%)	9 (4.35%)
-Lead/pocket erosion, *n* (%)	8 (3.86%)
-Ineffective therapy, *n* (%)	8 (3.86%)
-Necessity for pacing, *n* (%)	7 (3.38%)
-Lead rupture, *n* (%)	5 (2.42%)
-Defibrillation threshold testing failure, *n* (%)	5 (2.42%)
-Patient discomfort, *n* (%)	5 (2.42%)
-Lead malposition, *n* (%)	4 (1.93%)
-Technical issues, *n* (%)	2 (0.97%)
-Reel syndrome, *n* (%)	1 (0.48%)
-Necessity for MRI, *n* (%)	1 (0.48%)
-Premature battery depletion, *n* (%)	1 (0.48%)
Infective (pocket or lead), *n* (%)	80 (38.65%)
Not specified, *n* (%)	3 (1.46%)

CRT, cardiac resynchronization therapy; LVAD, left ventricular assist device; MRI, magnetic resonance imaging.

**Table 3:** Methods used for SLE.

Method Used for SLE	Total Population, *n* = 207
Manual traction, *n* (%)	99 (47.83%)
Additional incisions, *n* (%)	11 (5.31%)
Tools (sheaths), *n* (%)	14 (6.76%)
Not reported, *n* (%)	83 (40.10%)

SLE, S-ICD lead extraction.

**Table 4:** Management after SLE.

Management after SLE	Total Population, *n* = 207
ICD reimplantation, *n* (%)	102 (49.28%)
-S-ICD reimplantation, *n* (%)	25 (12.08%) *
-TV-ICD reimplantation, *n* (%)	75 (36.23%)
-Unspecified ICD reimplantation, *n* (%)	1 (0.48%)
-Scheduled for S-ICD, *n* (%)	1 (0.48%)
Heart transplantation, *n* (%)	7 (3.38%)
Medically managed, *n* (%)	1 (0.48%)
Declined reimplantation, *n* (%)	13 (6.28%)
Not known, *n* (%)	84 (40.58%)
-Lost to follow-up, *n* (%)	5 (2.42%)
-Not reported, *n* (%)	79 (38.16%)

ICD, subcutaneous implantable cardioverter defibrillator; TV-ICD, transvenous implantable cardioverter defibrillator. * 1 pt was reimplanted twice.

## Data Availability

No new data were created or analyzed in this study. Data sharing is not applicable to this article.

## Supplementary Materials

The following supporting information can be downloaded at: https://www.mdpi.com/article/10.3390/jcm12113710/s1. Supplementary Material: Quality Assessment.
