# Supplementary material for: Systematic Review on S-ICD Lead Extraction
Source: J Clin Med. 2023 May 27;12(11):3710. doi: 10.3390/jcm12113710 (PMC10254059; doi:10.3390/jcm12113710)
Supplement: Supplementary file 1 [file jcm-12-03710-s001.zip › jcm-2390484-supplementary.pdf]

QUALITY ASSESSMENT

- Gold et al. 2022 [14]

Question	Yes	No	Unclear	Not applicable
1. Were there clear criteria for inclusion in the case series?	X			
2. Was the condition measured in a standard, reliable way for all participants included in the case series?	X			
3. Were valid methods used for identification of the condition for all participants included in the case series?	X			
4. Did the case series have consecutive inclusion of participants?	X			
5. Did the case series have complete inclusion of participants?	X			
6. Was there clear reporting of the demographics of the participants in the study?			X	
7. Was there clear reporting of clinical information of the participants?			X	
8. Were the outcomes or follow-up results of cases clearly reported?			X	
9. Was there clear reporting of the presenting sites'/clinics' demographic information?			X	
10. Was statistical analysis appropriate?				X

- Giacomin et al. 2022 [15]

Question	Yes	No	Unclear	Not applicable
1. Were there clear criteria for inclusion in the case series?	X			
2. Was the condition measured in a standard, reliable way for all participants included in the case series?	X			
3. Were valid methods used for identification of the condition for all participants included in the case series?	X			
4. Did the case series have consecutive inclusion of participants?	X			
5. Did the case series have complete inclusion of participants?	X			
6. Was there clear reporting of the demographics of the participants in the study?			X	
7. Was there clear reporting of clinical information of the participants?			X	
8. Were the outcomes or follow-up results of cases clearly reported?			X	
9. Was there clear reporting of the presenting sites'/clinics' demographic information?	X			
10. Was statistical analysis appropriate?				X

- Russo et al. 2022 [16]

Question	Yes	No	Unclear	Not applicable
1. Were there clear criteria for inclusion in the case series?	X			
2. Was the condition measured in a standard, reliable way for all participants included in the case series?	X			
3. Were valid methods used for identification of the condition for all participants included in the case series?	X			
4. Did the case series have consecutive inclusion of participants?	X			
5. Did the case series have complete inclusion of participants?	X			
6. Was there clear reporting of the demographics of the participants in the study?			X	
7. Was there clear reporting of clinical information of the participants?			X	
8. Were the outcomes or follow-up results of cases clearly reported?			X	
9. Was there clear reporting of the presenting sites'/clinics' demographic information?	X			
10. Was statistical analysis appropriate?				X

- **Pothineni et al. 2022 [17]**

Question	Yes	No	Unclear	Not applicable
1. Were there clear criteria for inclusion in the case series?	X			
2. Was the condition measured in a standard, reliable way for all participants included in the case series?	X			
3. Were valid methods used for identification of the condition for all participants included in the case series?	X			
4. Did the case series have consecutive inclusion of participants?	X			
5. Did the case series have complete inclusion of participants?	X			
6. Was there clear reporting of the demographics of the participants in the study?	X			
7. Was there clear reporting of clinical information of the participants?	X			
8. Were the outcomes or follow-up results of cases clearly reported?	X			
9. Was there clear reporting of the presenting sites'/clinics' demographic information?	X			
10. Was statistical analysis appropriate?	X			

- **Migliore et al. 2021 [10]**

Question	Yes	No	Unclear	Not applicable
1. Were there clear criteria for inclusion in the case series?				X
2. Was the condition measured in a standard, reliable way for all participants included in the case series?				X
3. Were valid methods used for identification of the condition for all participants included in the case series?				X
4. Did the case series have consecutive inclusion of participants?				X
5. Did the case series have complete inclusion of participants?				X
6. Was there clear reporting of the demographics of the participants in the study?	X			
7. Was there clear reporting of clinical information of the participants?	X			
8. Were the outcomes or follow-up results of cases clearly reported?	X			
9. Was there clear reporting of the presenting sites'/clinics' demographic information?	X			
10. Was statistical analysis appropriate?				X

- **Allison et al. 2021 [20]**

Question	Yes	No	Unclear	Not applicable
1. Were there clear criteria for inclusion in the case series?				X
2. Was the condition measured in a standard, reliable way for all participants included in the case series?				X
3. Were valid methods used for identification of the condition for all participants included in the case series?				X
4. Did the case series have consecutive inclusion of participants?				X
5. Did the case series have complete inclusion of participants?				X
6. Was there clear reporting of the demographics of the participants in the study?	X			
7. Was there clear reporting of clinical information of the participants?	X			
8. Were the outcomes or follow-up results of cases clearly reported?	X			
9. Was there clear reporting of the presenting sites'/clinics' demographic information?	X			
10. Was statistical analysis appropriate?				X

- **Chung et al. 2021 [22]**

Question	Yes	No	Unclear	Not applicable
1. Were there clear criteria for inclusion in the case series?	X			
2. Was the condition measured in a standard, reliable way for all participants included in the case series?	X			
3. Were valid methods used for identification of the condition for all participants included in the case series?	X			
4. Did the case series have consecutive inclusion of participants?	X			
5. Did the case series have complete inclusion of participants?	X			
6. Was there clear reporting of the demographics of the participants in the study?			X	
7. Was there clear reporting of clinical information of the participants?			X	
8. Were the outcomes or follow-up results of cases clearly reported?			X	
9. Was there clear reporting of the presenting sites'/clinics' demographic information?	X			
10. Was statistical analysis appropriate?				X

- **Van der Stuijt et al. 2021 [23]**

Question	Yes	No	Unclear	Not applicable
1. Were there clear criteria for inclusion in the case series?	X			
2. Was the condition measured in a standard, reliable way for all participants included in the case series?	X			
3. Were valid methods used for identification of the condition for all participants included in the case series?	X			
4. Did the case series have consecutive inclusion of participants?	X			
5. Did the case series have complete inclusion of participants?	X			
6. Was there clear reporting of the demographics of the participants in the study?			X	
7. Was there clear reporting of clinical information of the participants?			X	
8. Were the outcomes or follow-up results of cases clearly reported?			X	
9. Was there clear reporting of the presenting sites'/clinics' demographic information?	X			
10. Was statistical analysis appropriate?				X

- **Gutleben et al. 2020 [24]**

Question	Yes	No	Unclear	Not applicable
1. Were there clear criteria for inclusion in the case series?				X

2. Was the condition measured in a standard, reliable way for all participants included in the case series?				X
3. Were valid methods used for identification of the condition for all participants included in the case series?				X
4. Did the case series have consecutive inclusion of participants?				X
5. Did the case series have complete inclusion of participants?				X
6. Was there clear reporting of the demographics of the participants in the study?	X			
7. Was there clear reporting of clinical information of the participants?	X			
8. Were the outcomes or follow-up results of cases clearly reported?	X			
9. Was there clear reporting of the presenting sites'/clinics' demographic information?	X			
10. Was statistical analysis appropriate?				X

- **Mitacchione et al. 2020 [25]**

Question	Yes	No	Unclear	Not applicable
1. Were there clear criteria for inclusion in the case series?				X
2. Was the condition measured in a standard, reliable way for all participants included in the case series?				X
3. Were valid methods used for identification of the condition for all participants included in the case series?				X
4. Did the case series have consecutive inclusion of participants?				X
5. Did the case series have complete inclusion of participants?				X
6. Was there clear reporting of the demographics of the participants in the study?	X			
7. Was there clear reporting of clinical information of the participants?	X			
8. Were the outcomes or follow-up results of cases clearly reported?	X			
9. Was there clear reporting of the presenting sites'/clinics' demographic information?	X			
10. Was statistical analysis appropriate?				X

- **Behar et al. 2020 [26]**

Question	Yes	No	Unclear	Not applicable
1. Were there clear criteria for inclusion in the case series?	X			
2. Was the condition measured in a standard, reliable way for all participants included in the case series?	X			

3. Were valid methods used for identification of the condition for all participants included in the case series?	X			
4. Did the case series have consecutive inclusion of participants?	X			
5. Did the case series have complete inclusion of participants?	X			
6. Was there clear reporting of the demographics of the participants in the study?	X			
7. Was there clear reporting of clinical information of the participants?	X			
8. Were the outcomes or follow-up results of cases clearly reported?	X			
9. Was there clear reporting of the presenting sites'/clinics' demographic information?	X			
10. Was statistical analysis appropriate?	X			

- **Patel et al. 2020 [28]**

Question	Yes	No	Unclear	Not applicable
1. Were there clear criteria for inclusion in the case series?				X
2. Was the condition measured in a standard, reliable way for all participants included in the case series?				X
3. Were valid methods used for identification of the condition for all participants included in the case series?				X
4. Did the case series have consecutive inclusion of participants?				X
5. Did the case series have complete inclusion of participants?				X
6. Was there clear reporting of the demographics of the participants in the study?	X			
7. Was there clear reporting of clinical information of the participants?	X			
8. Were the outcomes or follow-up results of cases clearly reported?	X			
9. Was there clear reporting of the presenting sites'/clinics' demographic information?	X			
10. Was statistical analysis appropriate?				X

- **Noel et al. 2020 [30]**

Question	Yes	No	Unclear	Not applicable
1. Were there clear criteria for inclusion in the case series?	X			
2. Was the condition measured in a standard, reliable way for all participants included in the case series?	X			
3. Were valid methods used for identification of the condition for all participants included in the case series?	X			
4. Did the case series have consecutive inclusion of participants?	X			
5. Did the case series have complete inclusion of participants?	X			
6. Was there clear reporting of the demographics of the participants in the study?	X			
7. Was there clear reporting of clinical information of the participants?	X			
8. Were the outcomes or follow-up results of cases clearly reported?	X			
9. Was there clear reporting of the presenting sites'/clinics' demographic information?	X			
10. Was statistical analysis appropriate?				X

- **Schaller et al. 2019 [31]**

Question	Yes	No	Unclear	Not applicable
1. Were there clear criteria for inclusion in the case series?	X			
2. Was the condition measured in a standard, reliable way for all participants included in the case series?	X			
3. Were valid methods used for identification of the condition for all participants included in the case series?	X			
4. Did the case series have consecutive inclusion of participants?	X			
5. Did the case series have complete inclusion of participants?	X			
6. Was there clear reporting of the demographics of the participants in the study?			X	
7. Was there clear reporting of clinical information of the participants?			X	
8. Were the outcomes or follow-up results of cases clearly reported?			X	
9. Was there clear reporting of the presenting sites'/clinics' demographic information?	X			
10. Was statistical analysis appropriate?				X

- **Migliore et al. 2019 [32]**

Question	Yes	No	Unclear	Not applicable
1. Were there clear criteria for inclusion in the case series?	X			
2. Was the condition measured in a standard, reliable way for all participants included in the case series?	X			
3. Were valid methods used for identification of the condition for all participants included in the case series?	X			
4. Did the case series have consecutive inclusion of participants?			X	
5. Did the case series have complete inclusion of participants?			X	
6. Was there clear reporting of the demographics of the participants in the study?			X	
7. Was there clear reporting of clinical information of the participants?			X	
8. Were the outcomes or follow-up results of cases clearly reported?			X	
9. Was there clear reporting of the presenting sites'/clinics' demographic information?			X	
10. Was statistical analysis appropriate?				X

- **Ip 2019 [11]**

Question	Yes	No	Unclear	Not applicable
1. Were there clear criteria for inclusion in the case series?				X
2. Was the condition measured in a standard, reliable way for all participants included in the case series?				X
3. Were valid methods used for identification of the condition for all participants included in the case series?				X
4. Did the case series have consecutive inclusion of participants?				X
5. Did the case series have complete inclusion of participants?				X
6. Was there clear reporting of the demographics of the participants in the study?	X			
7. Was there clear reporting of clinical information of the participants?	X			
8. Were the outcomes or follow-up results of cases clearly reported?	X			
9. Was there clear reporting of the presenting sites'/clinics' demographic information?	X			
10. Was statistical analysis appropriate?				X

- **Migliore et al. 2019 [33]**

Question	Yes	No	Unclear	Not applicable
1. Were there clear criteria for inclusion in the case series?	X			
2. Was the condition measured in a standard, reliable way for all participants included in the case series?	X			
3. Were valid methods used for identification of the condition for all participants included in the case series?	X			
4. Did the case series have consecutive inclusion of participants?	X			
5. Did the case series have complete inclusion of participants?	X			
6. Was there clear reporting of the demographics of the participants in the study?			X	
7. Was there clear reporting of clinical information of the participants?			X	
8. Were the outcomes or follow-up results of cases clearly reported?			X	
9. Was there clear reporting of the presenting sites'/clinics' demographic information?	X			
10. Was statistical analysis appropriate?				X

- **Orgeron et al. 2018 [34]**

Question	Yes	No	Unclear	Not applicable
1. Were there clear criteria for inclusion in the case series?	X			

2. Was the condition measured in a standard, reliable way for all participants included in the case series?	X			
3. Were valid methods used for identification of the condition for all participants included in the case series?	X			
4. Did the case series have consecutive inclusion of participants?			X	
5. Did the case series have complete inclusion of participants?			X	
6. Was there clear reporting of the demographics of the participants in the study?			X	
7. Was there clear reporting of clinical information of the participants?			X	
8. Were the outcomes or follow-up results of cases clearly reported?			X	
9. Was there clear reporting of the presenting sites'/clinics' demographic information?			X	
10. Was statistical analysis appropriate?				X

- **Viani et al. 2019 [35]**

Question	Yes	No	Unclear	Not applicable
1. Were there clear criteria for inclusion in the case series?	X			
2. Was the condition measured in a standard, reliable way for all participants included in the case series?	X			
3. Were valid methods used for identification of the condition for all participants included in the case series?	X			
4. Did the case series have consecutive inclusion of participants?	X			
5. Did the case series have complete inclusion of participants?	X			
6. Was there clear reporting of the demographics of the participants in the study?			X	
7. Was there clear reporting of clinical information of the participants?			X	
8. Were the outcomes or follow-up results of cases clearly reported?			X	
9. Was there clear reporting of the presenting sites'/clinics' demographic information?			X	
10. Was statistical analysis appropriate?				X

- **Nakhla et al. 2018 [36]**

Question	Yes	No	Unclear	Not applicable
1. Were there clear criteria for inclusion in the case series?	X			
2. Was the condition measured in a standard, reliable way for all participants included in the case series?	X			

3. Were valid methods used for identification of the condition for all participants included in the case series?	X			
4. Did the case series have consecutive inclusion of participants?	X			
5. Did the case series have complete inclusion of participants?	X			
6. Was there clear reporting of the demographics of the participants in the study?	X			
7. Was there clear reporting of clinical information of the participants?	X			
8. Were the outcomes or follow-up results of cases clearly reported?	X			
9. Was there clear reporting of the presenting sites'/clinics' demographic information?	X			
10. Was statistical analysis appropriate?	X			

- **Quast et al. 2018 [37]**

Question	Yes	No	Unclear	Not applicable
1. Were there clear criteria for inclusion in the case series?	X			
2. Was the condition measured in a standard, reliable way for all participants included in the case series?	X			
3. Were valid methods used for identification of the condition for all participants included in the case series?	X			
4. Did the case series have consecutive inclusion of participants?	X			
5. Did the case series have complete inclusion of participants?	X			
6. Was there clear reporting of the demographics of the participants in the study?			X	
7. Was there clear reporting of clinical information of the participants?			X	
8. Were the outcomes or follow-up results of cases clearly reported?			X	
9. Was there clear reporting of the presenting sites'/clinics' demographic information?	X			
10. Was statistical analysis appropriate?				X

- **Sponder et al. 2018 [38]**

Question	Yes	No	Unclear	Not applicable
1. Were there clear criteria for inclusion in the case series?	X			
2. Was the condition measured in a standard, reliable way for all participants included in the case series?	X			
3. Were valid methods used for identification of the condition for all participants included in the case series?	X			

4. Did the case series have consecutive inclusion of participants?			X	
5. Did the case series have complete inclusion of participants?		X		
6. Was there clear reporting of the demographics of the participants in the study?			X	
7. Was there clear reporting of clinical information of the participants?			X	
8. Were the outcomes or follow-up results of cases clearly reported?			X	
9. Was there clear reporting of the presenting sites'/clinics' demographic information?	X			
10. Was statistical analysis appropriate?				X

- **Clacaianu et al. 2017 [39]**

Question	Yes	No	Unclear	Not applicable
1. Were there clear criteria for inclusion in the case series?				X
2. Was the condition measured in a standard, reliable way for all participants included in the case series?				X
3. Were valid methods used for identification of the condition for all participants included in the case series?				X
4. Did the case series have consecutive inclusion of participants?				X
5. Did the case series have complete inclusion of participants?				X
6. Was there clear reporting of the demographics of the participants in the study?				X
7. Was there clear reporting of clinical information of the participants?	X			
8. Were the outcomes or follow-up results of cases clearly reported?	X			
9. Was there clear reporting of the presenting sites'/clinics' demographic information?	X			
10. Was statistical analysis appropriate?				X

- **Morani et al. 2017 [40]**

Question	Yes	No	Unclear	Not applicable
1. Were there clear criteria for inclusion in the case series?				X
2. Was the condition measured in a standard, reliable way for all participants included in the case series?				X
3. Were valid methods used for identification of the condition for all participants included in the case series?				X
4. Did the case series have consecutive inclusion of participants?				X
5. Did the case series have complete inclusion of participants?				X

6. Was there clear reporting of the demographics of the participants in the study?	X			
7. Was there clear reporting of clinical information of the participants?	X			
8. Were the outcomes or follow-up results of cases clearly reported?	X			
9. Was there clear reporting of the presenting sites'/clinics' demographic information?	X			
10. Was statistical analysis appropriate?				X

- **Frommeyer et al. 2016 [41]**

Question	Yes	No	Unclear	Not applicable
1. Were there clear criteria for inclusion in the case series?	X			
2. Was the condition measured in a standard, reliable way for all participants included in the case series?	X			
3. Were valid methods used for identification of the condition for all participants included in the case series?	X			
4. Did the case series have consecutive inclusion of participants?			X	
5. Did the case series have complete inclusion of participants?			X	
6. Was there clear reporting of the demographics of the participants in the study?			X	
7. Was there clear reporting of clinical information of the participants?			X	
8. Were the outcomes or follow-up results of cases clearly reported?			X	
9. Was there clear reporting of the presenting sites'/clinics' demographic information?	X			
10. Was statistical analysis appropriate?				X

- **Brouwer et al. 2016 [43]**

Question	Yes	No	Unclear	Not applicable
1. Were there clear criteria for inclusion in the case series?	X			
2. Was the condition measured in a standard, reliable way for all participants included in the case series?	X			
3. Were valid methods used for identification of the condition for all participants included in the case series?	X			
4. Did the case series have consecutive inclusion of participants?	X			
5. Did the case series have complete inclusion of participants?	X			
6. Was there clear reporting of the demographics of the participants in the study?			X	

7. Was there clear reporting of clinical information of the participants?			X	
8. Were the outcomes or follow-up results of cases clearly reported?			X	
9. Was there clear reporting of the presenting sites'/clinics' demographic information?	X			
10. Was statistical analysis appropriate?				X

- **Boersma et al. 2016 [44]**

Question	Yes	No	Unclear	Not applicable
1. Were there clear criteria for inclusion in the case series?	X			
2. Was the condition measured in a standard, reliable way for all participants included in the case series?	X			
3. Were valid methods used for identification of the condition for all participants included in the case series?	X			
4. Did the case series have consecutive inclusion of participants?			X	
5. Did the case series have complete inclusion of participants?			X	
6. Was there clear reporting of the demographics of the participants in the study?			X	
7. Was there clear reporting of clinical information of the participants?			X	
8. Were the outcomes or follow-up results of cases clearly reported?			X	
9. Was there clear reporting of the presenting sites'/clinics' demographic information?			X	
10. Was statistical analysis appropriate?				X

- **Frommeyer et al. 2015 [45]**

Question	Yes	No	Unclear	Not applicable
1. Were there clear criteria for inclusion in the case series?	X			
2. Was the condition measured in a standard, reliable way for all participants included in the case series?	X			
3. Were valid methods used for identification of the condition for all participants included in the case series?	X			
4. Did the case series have consecutive inclusion of participants?			X	
5. Did the case series have complete inclusion of participants?			X	
6. Was there clear reporting of the demographics of the participants in the study?	X			
7. Was there clear reporting of clinical information of the participants?	X			

8. Were the outcomes or follow-up results of cases clearly reported?	X			
9. Was there clear reporting of the presenting sites'/clinics' demographic information?	X			
10. Was statistical analysis appropriate?				X

- **Theuns et al. 2015 [49]**

Question	Yes	No	Unclear	Not applicable
1. Were there clear criteria for inclusion in the case series?	X			
2. Was the condition measured in a standard, reliable way for all participants included in the case series?	X			
3. Were valid methods used for identification of the condition for all participants included in the case series?	X			
4. Did the case series have consecutive inclusion of participants?	X			
5. Did the case series have complete inclusion of participants?	X			
6. Was there clear reporting of the demographics of the participants in the study?			X	
7. Was there clear reporting of clinical information of the participants?			X	
8. Were the outcomes or follow-up results of cases clearly reported?			X	
9. Was there clear reporting of the presenting sites'/clinics' demographic information?			X	
10. Was statistical analysis appropriate?				X

- **Jarman et al. 2013 [48]**

Question	Yes	No	Unclear	Not applicable
1. Were there clear criteria for inclusion in the case series?	X			
2. Was the condition measured in a standard, reliable way for all participants included in the case series?	X			
3. Were valid methods used for identification of the condition for all participants included in the case series?	X			
4. Did the case series have consecutive inclusion of participants?		X		
5. Did the case series have complete inclusion of participants?		X		
6. Was there clear reporting of the demographics of the participants in the study?			X	
7. Was there clear reporting of clinical information of the participants?			X	
8. Were the outcomes or follow-up results of cases clearly reported?			X	

9. Was there clear reporting of the presenting sites'/clinics' demographic information?			X	
10. Was statistical analysis appropriate?				X
